# Supplementary material for: COVID-19 and cause of pregnancy loss during the pandemic: A systematic review
Source: PLoS One. 2021 Aug 11;16(8):e0255994. doi: 10.1371/journal.pone.0255994 (PMC8357105; doi:10.1371/journal.pone.0255994)
Supplement: Attachment — Submitted filename: Response to reviewers-20210726.docx [file pone.0255994.s002.docx]

Dear Editor and reviewers,

Thank you for your considering our manuscript " COVID-19 and cause of pregnancy loss during the pandemic: A systematic review" We thank the editor and reviewers for their thoughtful critique and comments. We have carefully edited the manuscript as requested by reviewers and have provided a point-by-point response below. Please find the revised version included. We hope this meets the established reputation for the quality of your esteemed journal.

Thank you for considering our manuscript.

Corresponding author:

Mohammad Javad Nasiri, PhD, MPH

Email: [mj.nasiri@hotmail.com](mailto:mj.nasiri@hotmail.com)

**Reviewer #1**: Dear Respectable Editor

Thank you to give me the opportunity to review the article entitled (COVID-19 and cause of pregnancy loss during the pandemic: A systematic review)

The study designed to understand better the potential effects of Severe Acute Respiratory Syndrome Coronavirus 2 (SARS-CoV-2) on fetal loss in infected mothers presented with abortion following this infection.

The study concluded that there is an increased risk of abortion in mothers with a positive test result of SARS-CoV-2. Placental inflammation during the viral infection may result in fetal growth retardation and induce abortion. There has not been any consistent evidence of vertical transmission of the virus from mother to fetus, which requires further investigation.

Authors provided structured abstract and informative title

In the introduction, thy mentioned the controversy between studies regarding COVID-19 and explained why their study was designed (there is no systematic review reporting abortion in SARS-CoV-2 infected pregnant females).

In the methods section, they mentioned the search strategy, study selection, and data extraction and quality assessment.

Authors provided clear results, and a conclusion reflecting their results.

In addition, authors wrote the limitations of their study, future suggested research, and they used relevant references.

It my opinion its interesting study and can be accepted for publication without suggested changes from my side.

Regards

***Response:*** *Dear reviewer, thank you very much for your encouraging comments.*

**Reviewer #2**: Dear authors,

I appreciate your efforts to work on COVID-19. The manuscript is well written. Suggestions to improve:

1) Please add more data to “Introduction” section.

***Response:*** *Done as requested.*

2) Before using the abbreviation in main body of manuscript, please write it in full

***Response:*** *Done as requested.*

3) Please choose the keywords from the MeSH database.

***Response: Dear reviewer,*** when we searched PubMed and Embase, we used MeSH and Emtree. The manuscript was accordingly edited. (methods section, lines: 79-80).

4) Please add more data to “Discussion” to describe your valuable work.

***Response:*** *Done as requested.*
